# The Role of the Staphylococcal VraTSR Regulatory System on Vancomycin Resistance and *vanA* Operon Expression in Vancomycin-Resistant *Staphylococcus aureus*

**DOI:** 10.1371/journal.pone.0085873

**Published:** 2014-01-15

**Authors:** Nadia K. Qureshi, Shaohui Yin, Susan Boyle-Vavra

**Affiliations:** Section of Infectious Diseases, Department of Pediatrics, University of Chicago, Chicago, Illinois, United States of America; National Institutes of Health, United States of America

## Abstract

Vancomycin is often the preferred treatment for invasive methicillin-resistant *Staphylococcus aureus* (MRSA) infection. With the increase in incidence of MRSA infections, the use of vancomycin has increased and, as feared, isolates of vancomycin-resistant *Staphylococcus aureus* (VRSA) have emerged. VRSA isolates have acquired the entercoccal *vanA* operon contained on transposon (Tn) *1546* residing on a conjugal plasmid. VraTSR is a vancomycin and β-lactam-inducible three-component regulatory system encoded on the *S. aureus* chromosome that modulates the cell-wall stress response to cell-wall acting antibiotics. Mutation in *vraTSR* has shown to increase susceptibility to β-lactams and vancomycin in clinical VISA strains and in recombinant strain COLVA-200 which expresses a plasmid borne *vanA* operon. To date, the role of VraTSR in *vanA* operon expression in VRSA has not been demonstrated. In this study, the *vraTSR* operon was deleted from the first clinical VRSA strain (VRS1) by transduction with phage harvested from a USA300 *vraTSR* operon deletion strain. The absence of the *vraTSR* operon and presence of the *vanA* operon were confirmed in the transductant (VRS1Δvra) by PCR. Broth MIC determinations, demonstrated that the vancomycin MIC of VRS1Δvra (64 µg/ml) decreased by 16-fold compared with VRS1 (1024 µg/ml). The effect of the *vraTSR* operon deletion on expression of the van gene cluster (*vanA*, *vanX* and *vanR*) was examined by quantitative RT-PCR using relative quantification. A 2–5-fold decreased expression of the *vanA* operon genes occured in strain VRS1Δvra at stationary growth phase compared with the parent strain, VRS1. Both vancomycin resistance and vancomycin-induced expression of *vanA* and *vanR* were restored by complementation with a plasmid harboring the *vraTSR* operon. These findings demonstrate that expression in *S. aureus* of the horizontally acquired enterococcal *vanA* gene cluster is enhanced by the staphylococcal three-component cell wall stress regulatory system VraTSR, that is present in all *S. aureus* strains.

## Introduction

Invasive methicillin-resistant *Staphylococcus aureus* (MRSA) has emerged as a major public health problem, implicated in 18,000 deaths annually with an estimated 94, 360 invasive MRSA infections in 2005 [1]. Vancomycin is a glycopeptide antimicrobial agent that has been one of the most frequently used antibiotics for invasive MRSA infections. With the increased usage of vancomycin, there has been an increase in the number of MRSA isolates with reduced vancomycin susceptibility in the last decade [2]. According to the Clinical and Laboratory Standards Institute(CLSI), vancomycin-intermediate *S. aureus* (VISA) are those isolates with minimum inhibitory concentrations (MICs) between 4 µg/mL and 8 µg/mL, and vancomycin-resistant *S. aureus* (VRSA) are defined as those having MICs≥16 µg/mL [3].

The mechanism of vancomycin resistance in VRSA strains is entirely different from that of VISA strains. Whereas vancomycin intermediate resistance involves chromosomal point mutations and a thicker cell wall [4]–[6], VRSA isolates to date have acquired the *vanA* operon contained on transposon (Tn)1546 residing on a conjugal plasmid [7], [8]. *VanA* mediated resistance has been well studied in enterococci since the first *Enterococcus faecium* isolate with transmissible vancomycin resistance was reported in France in 1988 [9]. The *vanA* locus typically confers high-level vancomycin resistance (MICs 512–1024 µg/ml) to enterococcal species [10] by encoding the genes necessary for producing an altered peptidoglycan precursor in which the final dipeptide, D-alanyl-D-alanine (D-Ala-D-Ala) is replaced by depsipeptide, D-alanyl-D-lactate (D-Ala-D-Lac). Vancomycin binds with decreased affinity to this D-Ala-D-Lac terminus, thus rendering the bacteria resistant to the drug [11]. The *vanA* locus consists of seven genes, *vanRSHAXYZ*, whose expression is inducible by the glycopeptides, vancomycin and teicoplanin. Three of these genes (*vanHAX*) are necessary for production of the D-Ala-D-Lac containing peptidoglycan precursors [11]. VanR and VanS, encoded immediately upstream of *vanHAX*, comprise a two-component regulatory system responsible for the glycopeptide inducibility of *vanHAX* expression and the *vanSR* gene clusters. VanS is a membrane localized histidine kinase with an extracellular loop that has been proposed to be involved in sensing vancomycin leading to autophosphorylation of a conserved histidine residue [12]. This phosphoryl group is transferred to an aspartate in the cognate transcriptional activator, VanR, similar to other two component systems [13]. It has been shown in enterococci that upon induction with vancomcyin, the VanRS two-component system activates its own promoter and that of *vanHAX* leading to altered peptidoglycan precursors that confer resistance. VanY is a carboxypeptidase that is not necessary for resistance, but contributes to the resistance level [14]. VanZ confers resistance to teicoplanin by an unknown mechanism.

Of the 12 cases of VRSA reported in the USA, each has independently acquired the vancomycin-resistance transposon, Tn*1546*, independently from enterococcal donors [15]. Interestingly, *vanA*-containing *S. aureus* isolates exhibit a wide range of vancomycin MICs (vancomycin MIC 32 to 1024 µg/ml). We hypothesized that differential expression of native housekeeping genes amongst different *S. aureus* isolates could affect the phenotypic expression of acquired VanA-mediated vancomycin resistance.

Staphylococci have the ability to adapt quickly to antibiotic selection pressures resulting in development of resistant strains [16]. Exposure of antibiotics targeting the cell wall of *S. aureus*, activates the transcription of numerous genes encoding for cell-wall biosynthesis and metabolic pathways, known as the‘cell wall stress stimulon’ [17]–[20]. *VraTSR* is a vancomycin- and β-lactam-inducible three-component regulatory system that modulates a large proportion of genes comprising this cell-wall stress response of *S. aureus* [19]–[21]. The VraTSR regulatory system includes VraS, a sensor histidine kinase, and VraR a response regulator [22]. VraS and VraR are encoded together on a transcript downstream of two other genes, which we recently named *vraU* and *vraT* (previously called *yvqF*) [21]. VraT is necessary for methicillin resistance and for the activation of the VraTSR-dependent cell wall stimulon whereas *vraU* is not required for either of these activites [17], [21]. Thus, although *vraU* is encoded in the operon, we refer to the *vraTSR* operon since the role for *vraU* has not been found to date. Other studies have also shown that mutations in *vraTSR* genes can increase or decrease susceptibility to β-lactams and/or vancomycin in clinical MRSA and VISA strains [18], [19], [21], [22]. We reasoned that since the acquired enterococcal *vanA* operon encodes a heterologous cell wall biosynthesis gene cluster, MRSA strains might modulate *vanA* mediated vancomycin resistance by using VraTSR. To test this hypopthesis, we deleted the *vraTSR* operon from the first reported clinical VRSA strain and determined the effect on both vancomycin resistance and *vanA* operon expression.

## Materials and Methods

All experiments were conducted under Biosafety level 2 conditions with the approval of the Institutional Biosafety Committee at the University of Chicago.

### Bacterial strains and plasmids

The bacterial strains and plasmids used in this study are listed in Table 1. The VRSA parent strain used for deletion of the *vraTSR* operon was the first described clinical VRSA isolate carrying the Tn*1546*-borne *vanA* operon, VRS1 [7]. VRS1 was provided by the Network for Antimicrobial Resistance in *S. aureus* (NARSA) repository.

**Table 1:** Strains and Plasmids used in this study.

Strain or plasmid	Description	Source or reference
Strains		
VRS1	First clinical VRSA isolate from Michigan(Clonal cluster 5)	NARSA [8]
923 M23	A USA300 MRSA strain 923 with the *vraTSR* operon deletion (Clonal cluster 8)	[24]
VRS1Δvra	*vraTSR* operon deletion strain derived from strain 923	This study
VRS1cΔvra	VRS1Δvra deletion mutant complemented with a vra operon expressed on a low copy number plasmid pVRASR2 harboring the *vraTSR* operon; selectable by 10 µg/ml tetracycline	This study
Plasmids		
pVRASR2	Entire *vraTSR* operon cloned into pAW8	[22]

### Transduction

Propagation of phage and transduction of *S. aureus* was carried out according to standard procedures as described [23]. To produce a phage lysate, bacteriophage Φ 11, was propagated in the *vraTSR* deletion strain, 923-M23 which has all 4 genes of the *vraTSR* operon (*vraU*, *vraT*, *vraS*, and *vraR*) replaced with a *cat* gene as described [24]. The lysate was used to infect VRS1 at a multiplicity of infection of 1∶1 (phage-to-recipient). Transductants carrying a *vraTSR* operon deletion were selected on tryptic soy agar (TSA) supplemented with chloramphenicol at 10 µg/ml. As a control, the phage lysate was streaked alone to evaluate sterility and the possibility of reisolating the donor strain. VRS1cΔvra is a *vraTSR* operon deletion mutant complemented by all 4 genes in the *vraTSR* operon in a low copy number plasmid (pVRASR2) selectable by 5 or 10 µg/ml tetracycline as described [22].

### Effect of *vraTSR* deletion on growth

The growth of wild type strain VRS1, VRS1Δvra and complemented strain VRS1cΔvra were monitored using an incubated multi-mode plate reader (FLUOstar OPTIMA, BMG LABTECH) using conditions similar to an MIC assay. Briefly, the bacterial strains were grown overnight in tryptic soy agar (TSA) at 37°C. A colony from the overnight culture was inoculated in 0.9% saline and diluted to a cell concentration of 5×10^5^ CFU/ml in TSB or TSB supplemented with vancomycin at 32 µg/ml,512 µg/ml and 1 ml each was transferred to wells of a 48-well culture dish (Corning, Inc., Corning, NY) in quadruplicate and incubated at 37°C and the OD_600_ was measured every 20 min for 24 hrs. The plates were agitated by orbital shaking prior to each reading. Tetracycline was included in the wells containing VRS1c Δvra.

### Broth Minimum Inhibitory Concentrations (MIC) determinations

MICs were determined using the broth dilution method recommended by the Clinical and Laboratory Standards Institute (CLSI) [3] with exception of the use of BHI medium to optimize the vancomycin resistance phenotype as described previously [25]. An inoculum of 5×10^5^ CFU was applied to each well of a 24-well culture dish (Corning, Inc., Corning, NY) containing duplicates of two fold increasing concentrations of vancomycin from 0–1024 µg/ml. Oxacillin was tested at 0, 2, 4, 6, 12, 16, 24, 32, 48, 64, 96, 128 µg/ml. The dishes were incubated at 37°C and MICs were recorded at 24 hrs. Each MIC experiment was repeated at least 4 times.

### Growth conditions for evaluating the effect of *vraTSR* deletion on *van* gene expression

Since *vanA* and *vraTSR* expression are both inducible by vancomycin, two approaches were used to grow strains to evaluate the effect of the *vraTSR* deletion on expression of the *vanA* locus. In the first approach, we evaluated the steady state expression of *vanA* under continuous vancomycin inducing conditions. Strains were revived from frozen stocks stored in skim milk at −80°C onto TSA plates and incubated overnight at 37°C. The following day, a colony was inoculated into TSB supplemented with 2 µg/ml of vancomcyin to induce *vanA*, followed by incubation for 16 hours at 37°C with aeration. The next day, the overnight culture was diluted 1∶100 in fresh TSB, again with 2 µg/ml of vancomcyin to maintain expression of *vanA*. Bacteria were then harvested at midlog (OD_600_ of 0.5) and stationary (OD_600_ of 1.0) growth phases as assessed by spectrophotometer. (Bausch & Lomb; Spectronic 21)

The second approach evaluated the effect of a *vraTSR* deletion on induction of *vanA* expression shortly after exposure to vancomycin. To this end, a colony was inoculated into TSB and incubated for 16 hours at 37°C with aeration. The next day, the overnight culture was diluted 1∶100 in fresh TSB lacking vancomycin. When the culture reached an OD_600_ of 0.2, 2 µg/ml vancomycin was added to the medium. The RNA was harvested from the culture one hour later.

### RNA isolation and purification

At the desired times during growth, bacteria were pelleted by centrifugation and frozen at −80°C. To isolate RNA, the cells were thawed on ice, resuspended in the appropriate volume of TE buffer containing recombinant lysostaphin (Sigma, 1000 µg/mL) and incubated at room temperature for 10 mins to facilitate digestion of cell walls. The RNA was then extracted using the RNeasy kit (Qiagen) as directed by manufacturer's instructions, including treatment with DNase prior to RNA precipitation. The RNA concentration was determined from the optical density at 260 nm, and the quality was determined from the *A*_260_/*A*_280_ ratio and by analysis of rRNA using Agilent Bioanalyzer 2100.

### Quantitative real-time reverse transcription PCR (qRT-PCR) assay conditions

Reverse transcription was performed using 2 µg of total RNA using the High Capacity Archive cDNA Kit (Applied Biosystems) for cDNA synthesis. The real-time PCR was carried out using ABI 7500 Fast RT-PCR instrument. Prime Time primer design software was used to design primer/probe mixes for a 5′ nuclease assay from Integrated DNA Technologies (IDT). The qRT-PCR probes were each labeled at the 5′ end with the indicated fluorophore and were double quenched with internal ZEN and a Iowa Black® FQ at the 3′end. (Table 2) The concentration of each primer in qRT-PCR reactions was 500 nM whereas the probe concentration was 250 nM. Differences in gene expression were calculated by relative quantification(RQ) with the comparative ΔΔCt method [26] using the indicated reference strain as the comparator.

**Table 2:** Oligonucleotides and qRT-PCR probes used in this study.

Name	Sequence
**For qRT-PCR** a	
vanR	Forward: 5′-GTGGAGTAAAGGAGCAGAACG-3′
	Probe: 5′ 6-FAM/TTAATGACAAGGCCGGAGTGGACG-3′
	Reverse: 5′-GTTTTCACAGAGGATTCGCAG-3′
vanA	Forward: 5′-TTATAACCGTTCCCGCAGAC-3′
	Probe: 5′ 6-FAM/TTTGCCGTTTCCTGTATCCGTCCTC-3′
	Reverse- AAACATATCCACACGGGCTAG-3′
vanX	Forward: 5′-ATCGCATTGTAGGGACATACG-3′
	Probe: 5′ 6-FAM/AGTTGGCTGAATCGCTTTTGAAGGC-3′
	Reverse: 5′-AAGCAATCCGTACCCTTGG-3′
gyrB	Forward: 5′-AACGGACGTGGTATCCCAGTTGAT-3′
	Probe: 5′ Cy5/AAATGGGACGTCCAGCTGTCGAAGTT-3′
	Reverse: 5′-CCGCCAAATTTACCACCAGCATGT-3′
16S rRNA	Forward 5′-CAA TGG ACA ATA CAA AGG GCA G-3′
	Probe 5′ Cy5/CGC GAG GTC AAG CAA ATC CCA TAA AG 3′
	Reverse 5′-TGC AGA CTA CAA TCC GAA CTG-3′
For PCR	
vraS	Forward: 5′-ATGAACCACTACAATAG-3′
	Reverse: 5′-TTTAATCGTCATACGAATC-3′
vraR	Forward: 5′-ATGACGATTAAAGTATTG-3′
	Reverse: 5′-TTCGATACGAACTATTGA-3′
vanA	Forward: 5′- GGGAAACGACAATTGC-3′
	Reverse: 5′- GTACAATGCGGGCGTTA -3′

^a^ probes have 3′ Iowa Black Quencher and an an internal second quencher ZEN (IDT).

### Data Analysis

Relative quantitation of gene expression by qRT-PCR and MIC data were compared using Mann-Whitney test. All statistical data were analyzed by using Prism 5 program. (GraphPad Software, Inc., San Diego, CA). A *p-* value of ≤0.05 was considered significant.

## Results

### Characterization of growth of the *vraTSR* mutant and the mutant complemented with the *vraTSR* operon

**Figure 1:**
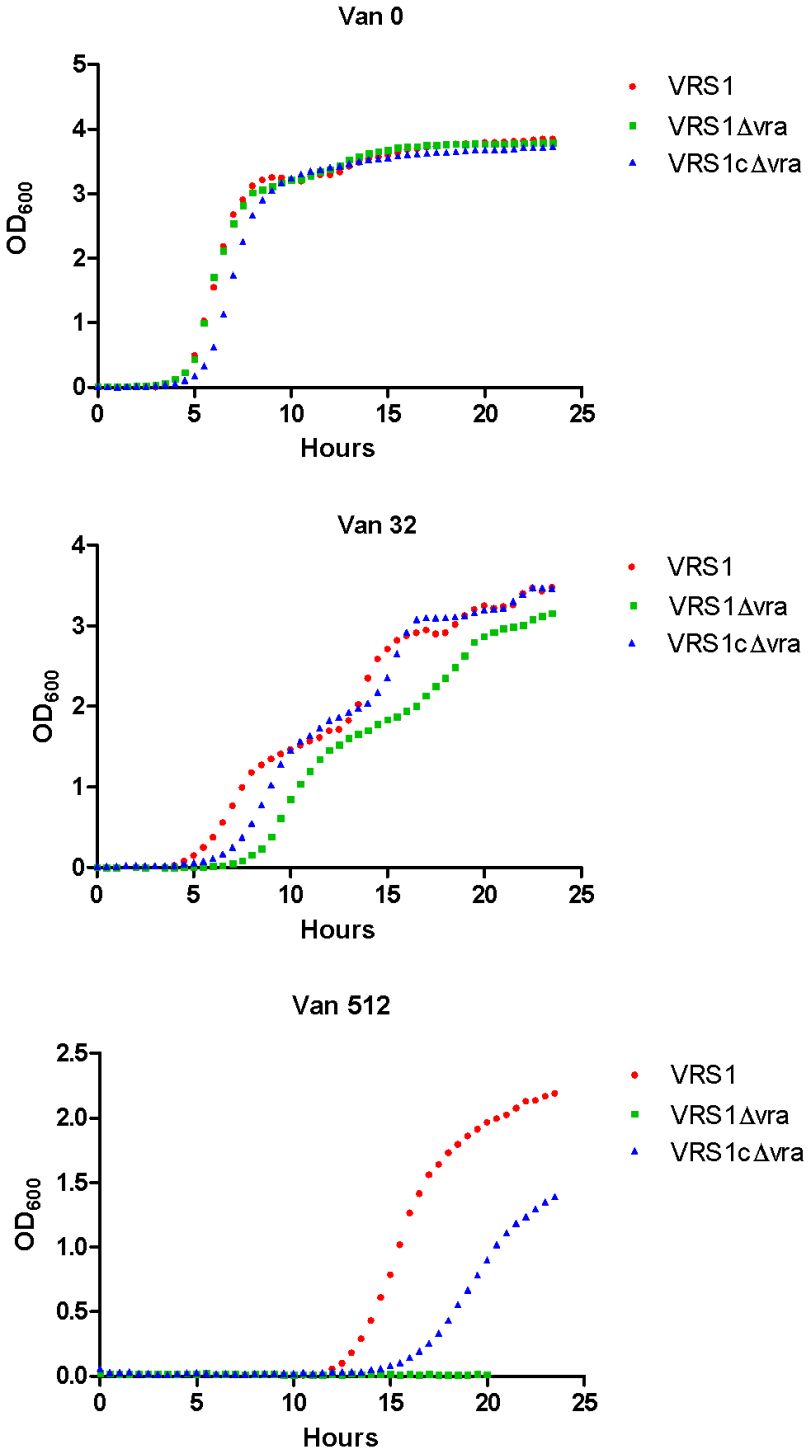
Comparison of VRS1, VRS1Δvra and VRS1 CΔvra growth curves in absence (Van0) and presence of vancomycin at concentrations of 32 µg/ml and 512 µg/ml (Van 32 and Van 512). Colonies were picked from TSA, diluted to a density equivalent to a 0.5 McFarland standard in 0.9% saline. This inoculum was diluted to 5×10^5^ CFU/ml and dispensed in a volume of 0.6 ml in each well.

The comparison of growth curves of wild type VRS1and the operon deletion strain VRS1Δvra demonstrate that deletion of *vraTSR* had minimal effect on fitness as shown by the similar growth curves of VRS1 and VRS1Δvra in absence of vancomycin (Fig. 1). At a subinhibiitory vancomycin concentration (32 µg/ml), the duration of the lag phase increased by about 3.5 hrs in strain VRS1Δvra compared with VRS1; however the growth rates of the two strains were similar in the presence of this amount of vancomycin. Complementation with the *vraTSR* operon decreased the duration of the lag phase of VRS1Δvra by 2 hrs when grown with 32 µg/ml of vancomycin, which is intermediate between the wildtype and mutant strains. The presence of 512 µg/ml of vancomycin increased the lag phase to over 10 hrs for strain VRS1 whereas strain VRS1Δvra did not grow. Growth of VRS1Δvra in 512 µg/ml of vancomycin was partially restored to that of the wildtype strain by complementation with the *vraTSR* operon in trans on a plasmid (strain VRS1cΔvra). These data demonstrate that at a sub-MIC of vancomycin, the *vraTSR* operon deletion has an effect on the lag phase rather than the growth rate but has little effect on fitness in the absence of vancomycin.

### Deletion of *vraTSR* decreased vancomycin resistance phenotype *in-vitro*

**Figure 2:**
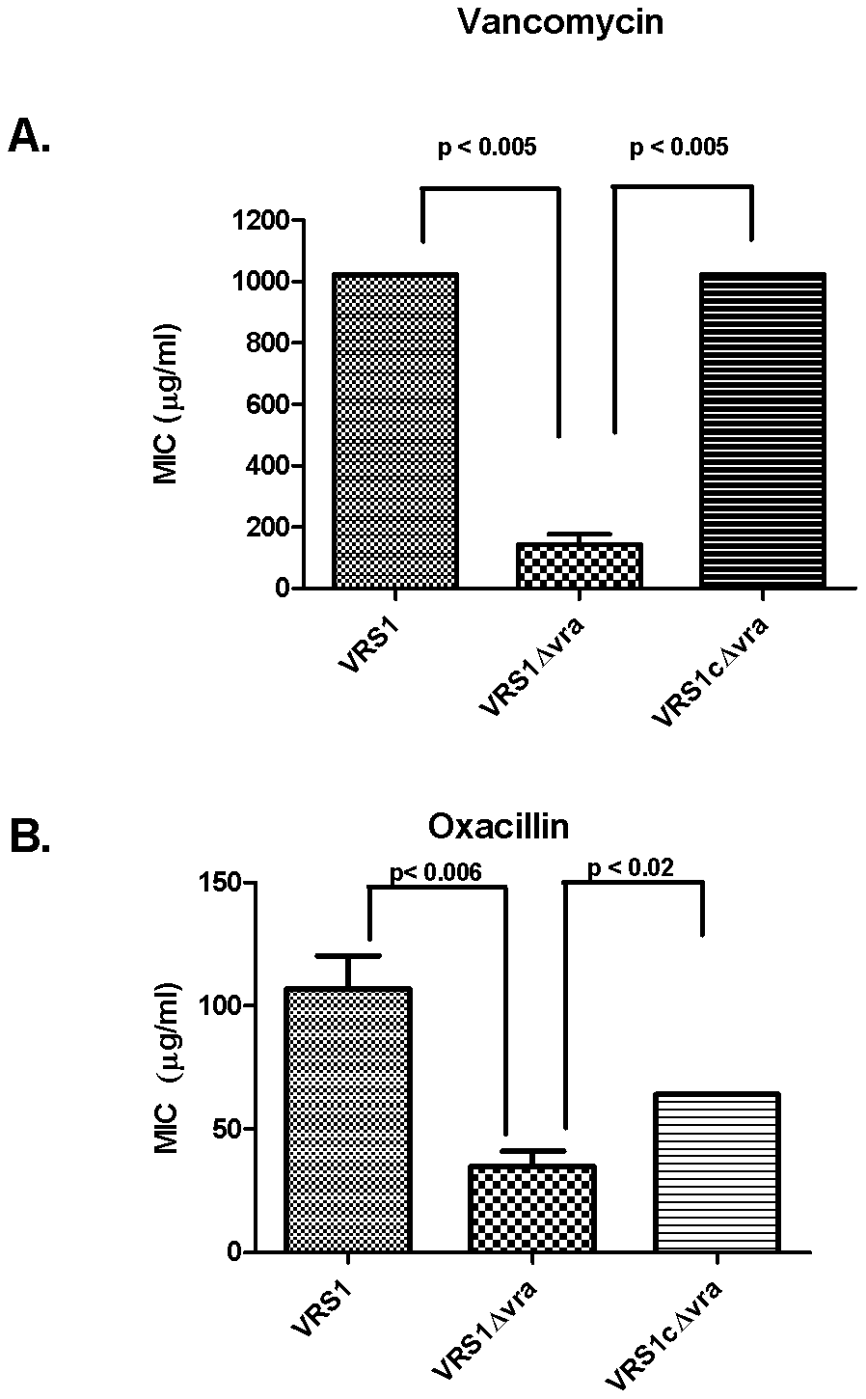
Comparison of MICs of the wild type VRS1, isogenic *vraTSR* deletion strain VRS1Δvra and complemented strain, VRS1 CΔvra. (A) MIC for Vancomycin (B) MIC for Oxacillin. The bars represent the SD of the mean MIC from at least 4 independent determinations. The lack of error bars indicates a SD of 0. Vancomycin was tested in BHI at 0, 8, 16, 32, 64, 128, 256, and 512 µg/ml. Oxacillin was tested in TSB containing 2% NaCl at 0, 8, 16, 32, 64 and 128 µg/ml; Tetracycline (5 µg/ml) was used to test strain VRS1cΔvra to maintain the complementation plasmid pVRASR2.

As expected from a previous study [7], the MIC of vancomycin for the clinical strain VRS1 at 24 hrs was 1024 µg/ml. Deleting the *vraTSR* operon from strain VRS1 significantly reduced resistance to vancomycin (mode MIC, 64 µg/ml) by 16 fold (p-value 0.0003)(Fig. 2A). Complementation of strain VRS1Δvra with the *vraTSR* operon, restored the vancomycin resistance phenotype to that of the wildtype.

### Deletion of *vraTSR* reduced resistance to oxacillin *in-vitro*

The VraTSR three component regulatory system has been shown to influence the methicillin resistance phenotype [18], [19], [21], [27]. Therefore, we determined the MIC of oxacillin of the mutant strain VRS1Δvra to assess the effect of the *vraTSR* deletion on methicillin resistance in a VRSA background. The MIC of oxacillin for the clinical strain VRS1 at 24 hrs was 128 µg/ml. Deleting the *vraTSR* operon from strain VRS1 significantly reduced resistance to oxacillin compared with the wildtype strain (MIC of 32 µg/ml, p-value<0.006) (Fig. 2B). The complementation of strain VRS1Δvra with the *vraTSR* operon expressed in trans increased the oxacillin MIC to 64 µg/ml. (p-value<0.03)

### Effect of deletion of *vraTSR* on steady state *vanA*, *vanX* and *vanR* expression (Figure 3)

**Figure 3:**
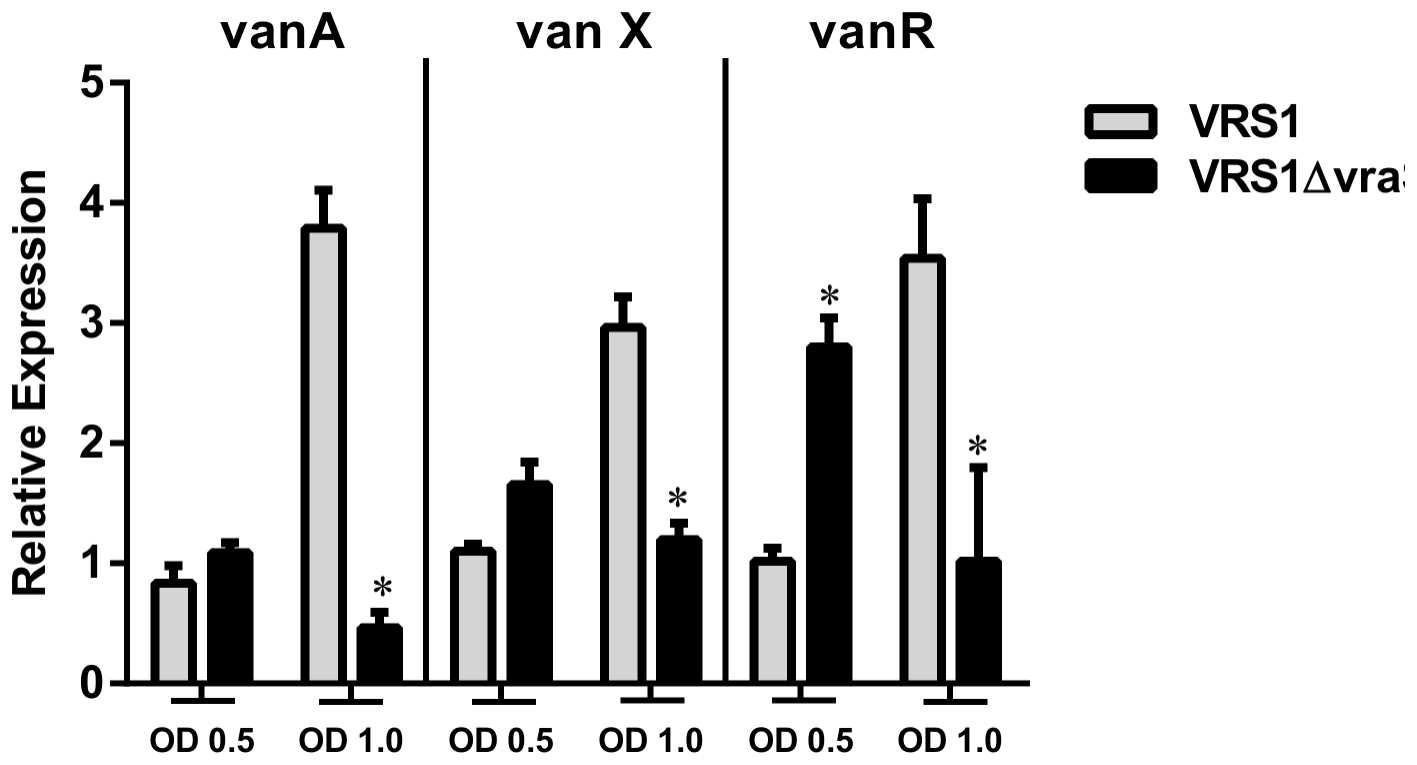
Effect of deletion of the *vraTSR* operon on steady state expression of the *vanA* operon genes by qRT-PCR. Subinhibitroy concentration of vancomycin (2 µg/ml) was present in the growth media in two successive passages to maintain constitutive expression of *vanA*. The cultures were harvested at midlog (OD_600_ 0.5) and stationary (OD_600_ 1.0) growth phases as described in Materials and Methods. Expression was quantified by relative quantification (RQ) using the comparative ΔΔCt method and the wild type strain VRS1 at midlog growth phase (OD_600_ 0.5) as the reference (after correction of each probe with an endogenous control). The *gyrB* gene probe labeled with the fluorophore Cy5 was used as the endogenous housekeeping gene and FAM-labeled probes for target genes *vanA*, *vanX* and *vanR* were used. The reactions were performed with three biological replicates with three technical replicates. * indicates p<0.05 compared with strain VRS1 at same time point. Error bars reflect propagated error calculated starting with the SD from the average C_T_ in each replicate.

Both the v*anA* and v*raTSR* operons are inducible by vancomycin [12], [22]. Thus we evaluated the effect of the *vraTSR* deletion on v*anA*, v*anX* and v*anR* expression under conditions that would ensure expression of *vanA* during growth. We first evaluated the effect of a *vraTSR* deletion on *vanA* operon expression during midlog and stationary growth phases. To maintain continuous steady state induction of *vanA*, subinhibitory vancomycin was present in the growth medium during an overnight passage and during the experiment. Expression of *vanA*, *vanX* and *vraR* were evaluated by qRT-PCR at midlog (OD_600_ of 0.5) and stationary (OD_600_ of 1.0) growth phases. (Fig. 3)

#### v*anA*

In strain VRS1, steady state expression of *vanA* increased from mid-log to stationary growth phase (4.7 fold, p 0.0004). In contrast, in VRS1Δvra, *vanA* expression decreased 2.1 fold from mid-log to stationary growth phase. Moreover, the difference of *vanA* expression was significantly lower at the stationary growth phase in VRS1Δvra compared with the wildtype strain (8.4 fold, p<0.0001).

#### v*anX*

Similar to *vanA*, steady state *vanX* expression in strain VRS1 increased from mid-log to stationary growth phase (3.1 fold, p 0.01). In contrast, in VRS1Δvra, *vanX* gene expression was similar in mid-log and stationary growth phases (1.2 fold, p 0.48). Moreover, *vanX* expression at stationary growth phase was significantly lower in VRS1Δvra compared with VRS1 (2.2 fold, p 0.002).

#### v*anR*

The steady state expression of *vanR* in strain VRS1 increased from mid-log to stationary growth phase (3.5 fold, p 0.004) as it did for *vanA* and *vanX*. In contrast, in VRS1Δvra, *vanR* expression precipitously dropped in stationary phase compared with the midlog phase by 8.8 fold (p<0.0001). At mid-log growth phase, a paradoxical effect was observed. The expression of *vanR* was higher in the deletion strain VRS1Δvra compared with VRS1 (2.6 fold, p 0.0004). In contrast, the expression of *vanA* or *vanX* was not significantly different at midlog phase between the wildtype and the mutant. Nevertheless, the significantly lower abundance of the *vanA* transcript in the *vraTSR* mutant compared with the VRS1 wildtype strain by the time cells reached stationary phase, is consistent with the findings for *vanA* and *vanX* and suggests that overall, expression of the *van* operon is lower in the *vraTSR* operon deletion strain.

### Deletion of *vraTSR* attenuates *vanA* and *vanR* gene induction by vancomycin (Fig. 4)

**Figure 4:**
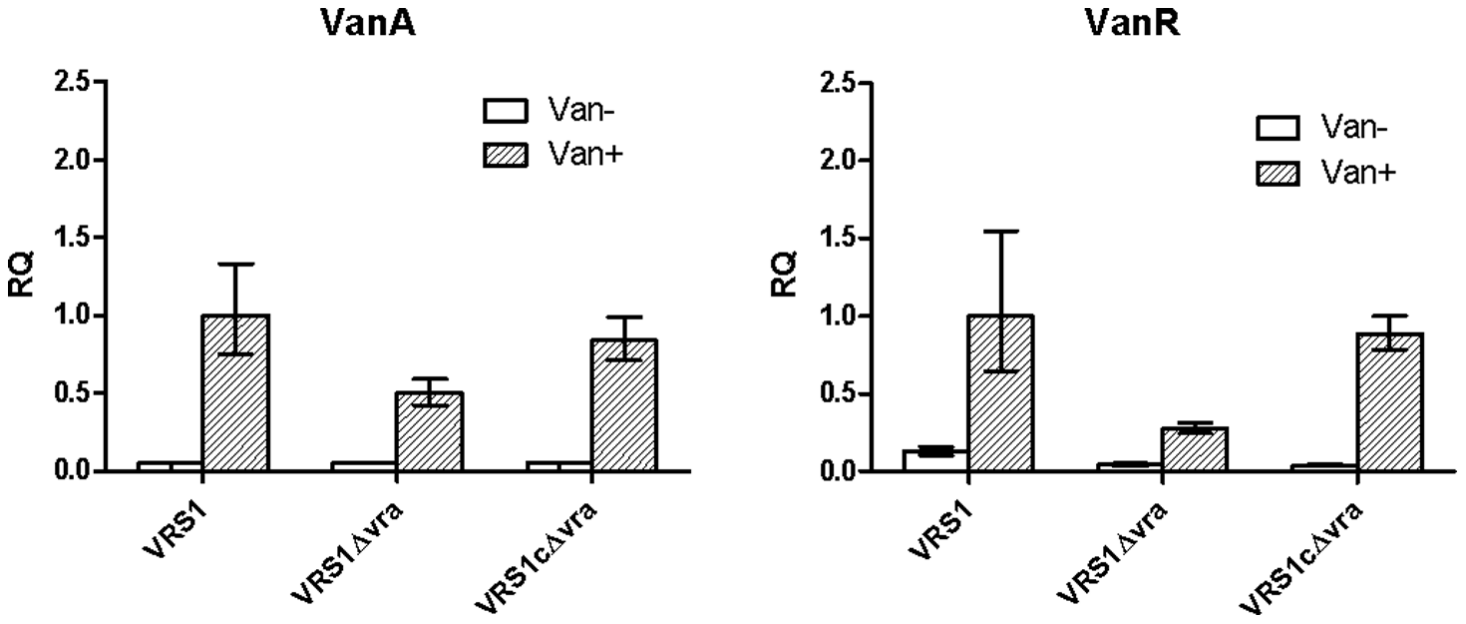
Effect of deletion of *vraTSR* on *vanA* and *vanR* gene induction by vancomycin. Expression of *vanA* operon genes in wildtype VRSA clinical strain (VRS1), the *vraTSR* deletion mutant (VRS1Δvra) and the *vraTSR*-complemented mutant (VRS1cΔvra), as measured by qRT-PCR. Vancomycin was added to early log cultures (when cultures reached an OD_600_ of 0.2) to induce *vanA* expression and RNA was isolated 1 hour later. Expression of each gene target was evaluated using relative quantification (RQ) with the comparative ΔΔCt method using strain VRS1 treated with vancomycin as the reference (after each probe was normalized to an endogenous control). Error bars reflect the range of RQ values from 3 experimental triplicates. The *vanA gene* probe was labeled with FAM and used in duplex reactions with the *16S* rRNA gene probe labeled with fluorophore Cy5 as the endogenous control. A FAM-labled *vanR* probe was used in a duplex reaction with a *gyrB probe* labeled *with* fluorophore Cy5 as the endogenous control. The choice of the endogenous controls were based on compatibility with the target in the duplex reaction.

We also examined the effect of *vraTSR* deletion on the induction of the *vanA* operon and whether the decreased expression of *vanA* in the *vraTSR* mutant could be complemented by overexpression of *vraTSR* in trans. To this end expression of *van* gene expression was evaluated 1 hr after addition of vancomycin to the medium in strains VRS1, VRS1Δvra and the *vraTSR* complemented mutant strain, VRS1cΔvra. As shown in Figure 4, expression of *vanA* and *vanR* were induced by vancomycin in all three strains but induction was attenuated in the *vraTSR* mutant. With restoration of *vraTSR* operon expression in the complemented strain, VRS1cΔvra, the expression of both *vanA* and *vanR* genes increased compared with the mutant VRS1Δvra and was comparable to the wild type, VRS1 (Fig. 4).

In cultures grown in the presence of vancomycin, the expression of *vanA* decreased two-fold in strain VRS1Δvra compared with the wildtype strain VRS1. Moreover, expression of *vanA* was restored to 84% of the wildtype control strain VRS1 in the *vraTSR*-complemented mutant, VRS1cΔvra, demonstrating that the loss of *vanA* induction in the *vraTSR* mutant involved VraTSR.

Comparing expression of *vanR* in the presence of vancomycin, there was a 3.6 fold decrease in *vanR* expression in strain VRS1Δvra compared with strain VRS1. *vanR* expression was restored to 88% of that of the wildtype in the presence of vancomycin in the complemented mutant VRS1cΔvra. *vanR* expression also decreased in the absence of vancomycin 2.9-fold in mutant strain VRS1Δvra compared with the wildtype strain. However, in the absence of vancomycin, *vraR* was expressed at the same level in both VRS1Δvra and VRS1cΔvra (Fig. 4). Thus, there was no complementation of *vanR* expression in the absence of vancomycin as there was in the presence of vancomyicn. This pattern was similar to that observed with *vanA* expression. This demonstrates that VraTSR has a greater influence on vancomyicn-dependent induction of *vanA* and *vanR* than it does on constitutive expression.

Although the deletion of *vraTSR* decreased expression of *vanR* and *vanA* in the presence of vancomycin, the fact that *vanR* and *vanA* expression did not diminish to that of the uninduced condition lacking vancomycin suggests that another factor besides VraTSR, such as VraR itself, is also involved in inducing *vanR* and *vanA* expression.

## Discussion

Vancomycin and other glycopeptides interfere with the terminal stages of peptidoglycan synthesis by forming a complex with the terminal D-Ala-D-Ala di-peptide of peptidoglycan precursors thereby preventing their incorporation with the growing peptidoglycan polymer. Horizontal acquisition of the *vanA* gene cluster results in vancomyin resistance by producing an alternative peptidoglycan precursor to replace the wildtype precursors [28]. But the van genes remain relatively silent unless the bacteria are exposed to a glycopeptide. Similarly, the native *vraTSR* regulatory system is designed to have increased expression in response to cell wall biosynthesis stress that is elicited by antibiotics such as vancomycin [19], [22], [29], oxacillin [18], [21], [27] or daptomycin [30]. Likewise *vraTSR* expression has been shown to increase in response to decreased expression of the native cell wall synthesis enzyme *pbp2* [18]. The result of upregulating the VraTSR system is a coordinated increase in expression of a regulon consisting of cell wall and metabolic genes that coordinately facilitate survival. The significance of this response is demonstrated by the fact that deletion or insertional mutagenesis of the *vraTSR* three-component regulatory system has been shown to increase the susceptibility of staphylococci to vancomycin, daptomycin and oxacillin in varying genetic backgrounds [18], [19], [21], [27], [29], [30].

This study provides further evidence for the important role of VraTSR in the adaptation to vancomycin, and shows for the first time that VraTSR plays a role in vancomycin resistance in clinical VRSA strains through regulation of *vanA* gene expression. This was demonstrated by an increased lag phase of growth at sub MIC of vancomycin in the *vraTSR* mutant and by the requirement of an intact *vraTSR* operon for maximal induction of *vanA* and *vanR* by vancomyin. Furthermore, we show that the increase in *vanA* operon gene expression from midlog to stationary growth phase is dependent upon an intact VraTSR cell wall stress sensing system. This suggests that *vanA* expression is induced as cells sense that growth is slowing. This phenomenon could be linked to a signal generated by increased autolysis and slowing of peptidoglycan precursor incorporation into the cell wall. It is worth noting that although prior studies have examined the inducibility of growth and D-Ala-D-Lac peptidoglycan precursor production in VRSA strains [31], this is the first study to examine *vanA* gene expression in *S. aureus*.

Previously, *vraTSR* has only been shown to influence expression of native staphylococcal genes. This study now shows that despite being a native gene encoded on the staphylococcal chromosome, *vraTSR* can be utilized by *S. aureus* to control the expression of heterologous cell wall biosynthesis operon that is acquired horizontally with the advantage of conferring antibiotic resistance. This represents a particularly clever strategy since both *van* operon expression and *vraTSR* are induced by vancomycin.

This study confirms and extends a prior study in which the effect of *vraTSR* on vancomycin resistance had been tested in a VRSA strain containing the *vanA* operon. Gardete et al. produced a strain COLVA_200_ΔvraS by introducing the plasmid from strain VRS1 into strain COL (a strain isolated in 1961) and deleting *vraTSR* [18]. In contrast, the approach taken in this study was to delete the *vraTSR* operon from the native clinical VRS1 strain which is the source of the plasmid used to construct COLVA_200_ΔvraS. Moreover, the strain used in our study belongs to the same clonal cluster as all other clinical VRSA isolates reported, clonal cluster 5 [32]. In contrast COLVA_200_ ΔvraS belongs to ST250 from clonal cluster 8. It was interesting that the *vraTSR* deletion in the clinical VRSA isolate decreased the vancomycin MIC to a greater extent (16-fold) than seen in the lab derived-strain (4-fold). Since both strains harbor the same *vanA* containing plasmid from VRS1, this provides evidence that factors in addition to *vraTSR* can account for differences in the level of *vanA* mediated vancomycin resistance among naturally occurring clinical isolates. This is consistent with historical data for oxacillin resistance. Although *vraTSR* affects resistance to oxacillin, strain specific factors other than *vraTSR* also influence the level of oxacillin resistance [33].

We observed a slight paradox on the effect of *vraTSR* during steady state *vanA* induction in midlog phase expression of *vanA* and *vanX* genes relative to *vanR* expression. Whereas midlog phase cultures of VRS1Δvra and VRS1 expressed similar levels of *vanA* and *vanX*, *vanR* expression was drastically higher in VRS1Δvra compared with VRS1. Nevertheless, at stationary phase *vanR* gene expression was drastically lower in the the *vraTSR* mutant compared with the wildtype, as it was for *vanA* and *vanX*.

Although vancomycin resistance decreases by 16 fold with a *vraTSR* operon deletion and is statistically significant, it may not be clinically important, as the vancomycin MIC still remains in the resistant range. It is possible however, that chemical inhibitors of VraTSR might be able to synergize with vancomycin to improve therapy of VRSA and VISA infections. This proof of principle remains to be tested in animal models of vancomycin therapy of *vraTSR* mutants, as we have done for oxacillin therapy of *vra* mutants of MRSA [21], [24].

The molecular mechanism by which VraTSR affects *vanA* operon expression remains to be determined. It is possible that there is cross talk between the two regulatory systems, VraTSR and VanRS. Indeed, it has been shown in *Enterococcus faecalis* that VanR is activated in the abscence of VanS by another histidine kinase [12]. It is also possible that one of the 40 genes that are activated by VraTSR in response to vancomycin is responsible for the activation of *vanA* by *VraTSR*. These possibilities will be explored in future studies.
